# Van der Woude Syndrome Associated with Hypodontia: A Rare Clinical Entity

**DOI:** 10.1155/2012/283946

**Published:** 2012-12-23

**Authors:** Romesh Soni, Rajul Vivek, Adit Srivastava, Ankita Singh, Shalabh Srivastava, T. P. Chaturvedi

**Affiliations:** ^1^Department of Prosthodontics, Faculty of Dental Sciences, Institute of Medical Sciences, Banaras Hindu University, Varanasi 221005, India; ^2^Department of Oral Medicine and Radiology, Faculty of Dental Sciences, IMS, Banaras Hindu University, Varanasi 221005, India; ^3^Department of Oral Pathology and Microbiology, Jaipur Dental College, Jaipur, Rajasthan 302020, India; ^4^Department of Orthodontics, Faculty of Dental Sciences, IMS, Banaras Hindu University, Varanasi 221005, India

## Abstract

Van der Woude syndrome (VWS) is usually underreported and frequently not diagnosed. The phenomenon that cleft lip and palate are regularly combined in the same pedigree makes it unique. A meticulous examination of a patient with lip pits may reveal a hidden form of a cleft, for example, submucous. This paper presents a case of VWS in a ten-year-old boy with characteristic orofacial features. Special emphasis has also been given on the need for appropriate genetic counseling.

## 1. Introduction 

Van der Woude syndrome (VWS) is a rare developmental, congenital malformation with autosomal dominant inheritance, high penetrance, and variable expressivity, occurring in about 1 of every 1,00,000–2,00,000 people [1]. Van der Woude was the first to combine lower lip pits with cleft lip (CL) and palate (CLP), introducing a new clinical entity, while she also described its mode of heredity. Van der Woude syndrome is characterized by pits and sinuses of lower lip, cleft lip with or without cleft palate. Sometimes lip pits may be the only manifestation of this syndrome. Missing incisors or premolars is a common finding. Other oral manifestations include congenital adhesion of the jaws, narrow high arched palate, and ankyloglossia. It was suggested that genetic defect of lip pits is due to microdeletion on chromosome bands 1q32-q41.10. Other anomalies beside oral manifestation like limb anomalies, popliteal webs, brain abnormalities, accessory nipples, congenital heart defects are also seen [2]. The following case report describes a case of VWS including pits in lower lip, cleft in upper lip and palate, and hypodontia. 

## 2. Case Report 

A ten-year-old boy came to Faculty of Dental Sciences, Institute of Medical Sciences, Banaras Hindu University, with chief complaint of nasal regurgitation during eating. He also wanted esthetic correction of his face. The family history did not reveal any sign of cleft lip and palate. On taking detailed history from his mother, it was revealed that the patient was born after an uneventful full-term pregnancy. There was no history of medication or illness of mother during pregnancy. Further, patient had undergone repair of bilateral cleft lip at age of ten months. 

**Figure 1:**
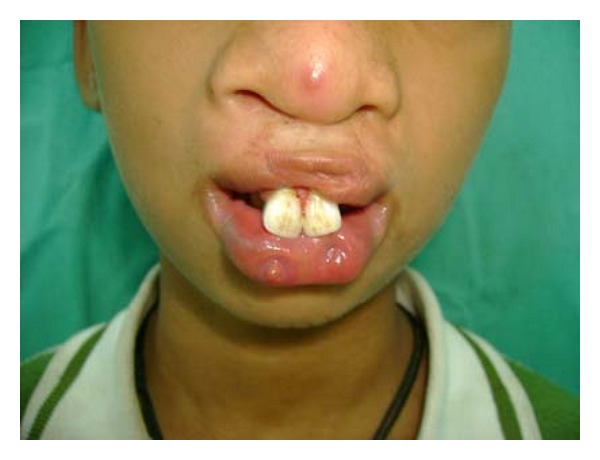
Maxillary central incisors hanging down along with premaxilla and bilateral pits in lower lip—frontal view.

**Figure 2:**
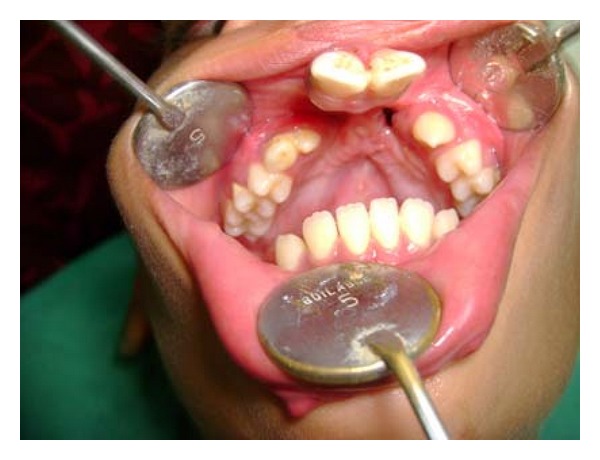
Intraoral view showing bilateral alveolar clefts in premaxillary region.

Extraoral examination revealed premaxilla along with maxillary central incisors hanging down giving unaesthetic appearance to the patient. The lower lip showed bilateral, paramedian small pits (Figure 1). Intraoral soft tissue examination showed bilateral alveolar clefts in premaxillary region (Figure 2). Further, the maxillary arch was constricted with high arch palate. Patient was having mixed dentition with congenital missing bilateral maxillary lateral incisor.

**Figure 3:**
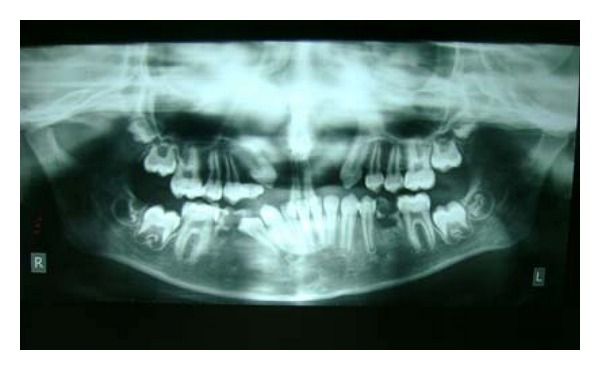
OPG disclosing missing bilateral maxillary lateral incisor tooth germ.

Patient also reported recurrent respiratory tract infections, breathlessness, regurgitation of food, and nasal twang during speaking. Radiographic examination from orthopantomograph (OPG) disclosed bilateral maxillary lateral incisor tooth germ was missing indicating hypodontia (Figure 3). Patient is planned for surgical correction of cleft lip and palate as well as minor cosmetic implications. Patient should undergo surgical correction. In the meanwhile he is instructed to keep meticulous oral hygiene care. 

## 3. Discussion 

Congenital pits of the lower lip constitute a rare developmental malformation, transmitted by autosomal dominant mode, with considerable heterogeneity as regards to the expression of the disorder. They are present in Van der Woude syndrome (VWS), in which clefts of the upper lip and palate are often observed. Lip pits are usually circular or oval but can also be transverse, slit-like, or sulci. The elevation may rarely fuse in midline, producing a snout-like structure [3]. Lower lip pits are usually asymptomatic, and the only symptom might be the continuous or intermittent drainage of watery or salivary secretions [4].

The prevalence of VWS varies from 1 : 100000 to 1 : 40000 still born or live births. No significant difference between sex is reported as regards to the prevalence of the syndrome [5]. Apart from the major signs, there are other features that are often associated with VWS patients with number of the teeth missing in the upper jaw almost double that in corresponding control groups. VWS patients may rarely show clefts without pits. These cases represent a small group of cleft patients with a high recurrence risk and underline the need for specific questions and examination for lip pits including microforms, in relatives of cleft patients at genetic counseling.

The treatment of VWS patients includes all necessary surgical and multidisciplinary procedures for the correction of serious anomalies including clefts. As far as the treatment of lower lip pits is concerned, spontaneous shrinkage has been reported in rare cases.

## 4. Conclusion

Although rare, Van der Woude syndrome (VWS) should be considered in the differential diagnosis of cleft lip and palate. Dentist may be the first person to diagnose this syndrome and therefore should be aware of it. Proper treatment plan at the correct time will help the dentist to satisfy the psychological and esthetic needs of the patients. Further, genetic counseling is highly recommended.
